# Relationship Difficulty Is Associated With Poorer Mental Health in Later Life

**DOI:** 10.1093/geronb/gbaf094

**Published:** 2025-05-23

**Authors:** Lea Ellwardt, Theo G van Tilburg

**Affiliations:** Department of Sociology and Social Psychology, University of Cologne, Cologne, Germany; Department of Sociology, Vrije Universiteit Amsterdam, Amsterdam, The Netherlands; (Psychological Sciences Section)

**Keywords:** Anxiety, Depression, Loneliness, Social networks

## Abstract

**Objectives:**

Integration into social networks is an important promotor of well-being and aging healthily, yet the dark side of social networks includes encounters with network members that are perceived as unpleasant, demanding, or difficult. This study investigates the association of relationship difficulty in older adults’ core networks with the mental health outcomes of loneliness, depression, and anxiety.

**Methods:**

Survey data were collected from the Longitudinal Aging Study Amsterdam (LASA) on relationship difficulty in personal networks and mental health. The sample included 892 respondents (mean age = 73; range = 61–100). The analytical models used 2-step estimation with inverse-probability weights for testing differences in the three mental outcomes between older adults with and without difficult relationships. Differences were tested using cross-sectional and longitudinal mental health observations.

**Results:**

The presence of relationship difficulty was significantly associated with poorer mental health on all three conditions in the cross-sectional models. Lagged effects were significant on depression only and weakest on anxiety.

**Discussion:**

For some older adults, instances where they feel pressured into upholding and continuing difficult relationships can be linked to higher incidences of adverse mental health outcomes. As such, social integration can be a double-edged sword, and research and practice should consider its potentially negative impacts.

The availability of supportive social relationships is an important asset in aging healthily. Older adults who are surrounded by a large and diverse number of social contacts experience fewer cardiovascular responses to stress, more positive psychological outcomes (Uchino et al., 2001), less cognitive decline (Ellwardt et al., 2014; Piolatto et al., 2022), and live longer (Ellwardt et al., 2015; Holt-Lunstad et al., 2010) than socially isolated older adults and those with restricted networks. Social networks provide a significant reserve for support when needs and dependency grow with age, and for enduring and bouncing back from acute crises (MacLeod et al., 2016).

Nevertheless, there is heterogeneity in the extent to which older adults may benefit from being embedded in social relationships. Specifically, some of this variance in benefit is explained by differences in whether network ties are perceived as having a positive, negative, or ambivalent quality (Uchino et al., 2001). Positive relationships are generally protective, but negative and ambivalent ones pose a health risk (Rook, 2015) because negative social exchanges increase the individual’s vulnerability to emotional distress (August et al., 2007). Crucially, the presence of a single difficult relationship may distract from other more supportive relationships, which seemingly makes it a particularly harmful stressor even in well-integrated older adults. Ambivalent relationships with a child and strain from a spouse have, for instance, been linked to elevated loneliness in adults aged 50 years and older (Chen & Feeley, 2014; Hua et al., 2021). Associations with health outcomes, however, are complex: On the one hand, relationship negativity has been linked to increased mortality among healthy adults aged 40 and above, suggesting a main effect; and on the other hand, research has shown decreased mortality among individuals with chronic illnesses, indicating a potential buffering effect (Birditt & Antonucci, 2008). In light of such findings, interest in difficult relationships has grown in recent years among both the general (Offer, 2021) and older populations (Cornwell & Schafer, 2016). Previous research predominantly focused on positive relationships and conflict within intergenerational (Girardin et al., 2018; Sapin et al., 2016) and care relationships (Martire et al., 2002; Newsom, 1999), with little attention toward determining a comprehensive set of mental health outcomes.

Against this background, we aim to extend previous research by identifying three related yet conceptually distinct mental health outcomes—loneliness, depression, and anxiety—in older adults based on their presence or lack of difficult relationships. We first cross-sectionally test the association between the presence of a difficult relationship and adverse mental health outcomes by assessing personal networks and mental health during one set of observations. Then, we test the longitudinal robustness of this association by reassessing mental health at a later time point during a collectively shared stressful life event: the COVID-19 pandemic, which contributed to declines in sociability and mental health (Van Tilburg et al., 2021; Völker, 2023).

Our study advances existing research designs and contributes to the literature on older adults’ personal networks and mental health in several ways. First, our sample from the Dutch population-based Longitudinal Aging Study Amsterdam (LASA) included individuals up to 100 years old, which was important in capturing the “old-olds,” those most susceptible to social isolation and loneliness (Luhmann & Hawkley, 2016). Second, our measure of relationship difficulty was not limited to a particular relationship domain (e.g., the family or care network) but was open to any social role. Third, we collected data on relationships with specific members in the core network using name generators instead of a generic scale, enabling us to obtain higher reliability in the independent variable. Fourth, the data included confounding variables on relationship difficulty among a respondent’s network members (i.e., quarrels between others not directly involving the respondent), which allowed us to single out the effect of the respondents’ personal difficult relationships. Finally, our analysis resembled an experimental design by testing three different mental health outcomes cross-sectionally (without a crisis) and longitudinally (during a crisis).

## Theory and Evidence

Difficult relationships are characterized by frequent negative encounters, including aversive and inconsiderate behaviors, such as demanding, judging, arguing, and fighting (Offer, 2021). Notably, while difficult relationships will consist of mostly negative and some positive encounters (Offer, 2021), ambivalent relationships contain an equal combination of negative and positive encounters (Ross et al., 2019). In our analysis, we merged ambivalent and negative relationships into one category of difficult relationships for two reasons, as described below.

First, purely negative relationships in personal networks are theoretically and empirically unlikely. People prefer connecting with supportive others and, where possible, avoid interaction partners that they find troublesome or irritating (Harrigan & Yap, 2017). This preference likely gets stronger in old age: According to socioemotional selectivity theory (Carstensen, 1992, 2021), as people age, they prioritize emotionally rewarding relationships, and when such relationships are present, having a smaller network does not negatively affect well-being. In line with this view, previous research on 50–70-year-old adults has shown a low prevalence of difficult relationships: Offer and Fischer (2018) found that about 5% of relationships with network members were reported as “difficult-only ties,” while the broader category of “difficult engaged-in-exchange ties” yielded a slightly higher prevalence of 8%, thus constituting a total of 13% of relationships that were “difficult ties.” Offer (2020) operationalized difficult relationships as interactions with others that respondents “sometimes find demanding or difficult” to facilitate a quantitative inquiry (Offer, 2020).

Second, difficult relationships tend to weigh disproportionally more heavily on well-being and mental health than positive ones (Rook, 2015). Cognitive psychologists explain this *negativity effect* as the brain attributing greater significance to negative than positive information (Ito et al., 1998), which leads to more intense evaluative and behavioral responses to threatening interactions within and across relationships. An implication for ambivalent relationships is that negative encounters may overshadow the positive encounters, thereby canceling out or even reversing any health-promoting effects (Lee & Szinovacz, 2016; Ross et al., 2019; Uchino et al., 2012). Similarly, when attention is drawn to threatening interaction partners, a single difficult relationship may overshadow any other positive relationships in the same network. Relationship difficulty in personal networks can thus present a significant stressor, even when most network members are perceived as pleasant. To better capture these mechanisms, our analysis focuses on the core network by selecting the most frequently contacted important ties, as these relationships are most relevant for daily interactions and well-being.

An important mechanism linking social relationships and health is the attenuation of harmful stressors (Thoits, 2011) that may arise from ongoing adversity or abrupt crises. This stress-buffering effect is considerably lower in unsupportive relationships and entirely reversed in negative social exchanges, the latter of which likely cause relationship stress and, as such, enhance health-undermining outcomes instead of buffering them (Umberson & Karas Montez, 2010). Related consequences that may add further strain to an individual’s relationships include the individual in a negative social exchange facing an increased susceptibility to loneliness, depression, and anxiety.

Relationship difficulty has been associated with more significant life stress (Rook, 2003), greater psychological distress (August et al., 2007; Offer, 2020), a higher likelihood of anxiety and mood disorders (Bertera, 2005; Lincoln et al., 2010), and lower self-rated health (Newsom et al., 2008). Furthermore, exposure to difficult relationships lowers satisfaction with one’s social network (Rook, 2003) and increases loneliness (Chen & Feeley, 2014; Fiori & Consedine, 2013; Offer, 2020). Within supportive relationships, negative reactions to care from a spouse have been associated with heightened depressive symptomatology (Martire et al., 2002; Newsom, 1999). We thus expect older adults in difficult relationships to cope less well than those without difficult relationships in their network, as predicted in the following hypothesis: *Individuals with relationship difficulty experience poorer mental health than individuals without relationship difficulty.* Here, as we assume similar mechanisms in times without and with crises, we expect both cross-sectional and longitudinal associations to be identified in our study.

Before detailing our research design, we wish to acknowledge the impact of the reverse mechanism whereby permanently distressed or socially anxious people will likely reinforce and create difficult social encounters themselves, risking the creation and propagation of a negativity spiral (Segel-Karpas & Ayalon, 2020). We note that the longitudinal design of our study limits but does not eliminate this possibility. To increase the validity of our study findings, we will test our hypothesis among three different mental health outcomes—loneliness, depression, and anxiety—rather than relying on a single one.

We also note that loneliness differs from depression and anxiety as such that there is no medical treatment. Yet, loneliness has been considered a public health problem and has been included in psychometric assessments of mental conditions like depression, which is why we refer to loneliness as a mental health outcome here. Finally, although the three concepts are related, they are conceptually distinct from both a psychological/treatment perspective and an empirical standpoint (loneliness, e.g., has been shown to lead to, but is stochastically distinguishable from, depressive symptomatology; Cacioppo et al., 2010). Loneliness is most directly influenced by social network quality but can also arise from a lack of positive ties rather than the presence or overrepresentation of difficult ones. Anxiety may stem from uncertainty about support and a perceived loss of control in social relationships, while depression or sadness may result from ongoing irritation, disappointment, or feeling let down. While our research design does not allow for a detailed examination of these underlying psychological mechanisms, we assume that multiple intertwined factors contribute to the observed association between relationship difficulty and overall mental health.

## Research Design

### Respondents

Data were obtained from the LASA (Huisman et al., 2011). Three random samples of men and women born between 1908 and 1957 were taken from the population registers of three cities and six surrounding small municipalities. After baseline observations were taken in 1992, 2002, and 2012, all respondents were interviewed every three or four years; attrition across waves is presented in Supplementary Figure 1 in Supplementary Material. In the current study, our starting point was the eighth follow-up observation conducted in 2018–2019 (*T*_1_; *N* = 1,591). Data collection consisted of three elements: a personal interview, a self-administered questionnaire, and a medical interview that included physical testing. For the current study, a second self-administered questionnaire was sent after approximately one month with additional questions about respondents’ personal networks, including items about difficulties in relationships. We also employed data from the COVID-19 Additional Study (*T*_2_; 2020), which was conducted one to one-and-a-half years after the previous two studies and was thus considered a longitudinal follow-up observation in our analysis. The LASA study, including all additional studies in this analysis, was conducted in line with the Declaration of Helsinki and received approval from the medical ethics committee of VU University Medical Center (IRB numbers: 92/138, 2002/141, 2012/361, and 2016/301).

### The *T*_1_ Interview

The LASA interview data include a wide variety of health variables, including several scales on physical, cognitive, and mental health. Importantly, this survey contains a particularly comprehensive module to assess respondents’ personal networks. That is, it used a domain-specific approach for network delineation according to the following classification of personal relationships: household members (including partners, if applicable), children and their partners, other family members, neighbors, contacts through work (including voluntary work), members of associations (e.g., sports clubs, churches, political parties), and other nonkin relationships (e.g., friends and acquaintances). For each of the seven domains, the following name-generating question was posed: “Name the people you have frequent contact with and who are also important to you” (Van Tilburg, 1998). Using this prompt of frequent contact (in general) served to generate names, and left the interpretation of what frequent contact is to the respondent. Specific contact frequency was reported in a subsequent step and showed that there was considerable variation in whether respondents had daily, weekly, or any other kind of contact with network members. Respondents could only name a person once; in practice, the number of names that could be given was unlimited. The criteria of importance were left to the interpretation of respondents, although they could only consider persons aged 18 and older. Notably, relationship difficulty was not measured in the interview.

### The *T*_1_ Additional Network Questionnaire

A self-administered questionnaire was sent to respondents who had generated at least two network members in the main interview; the questionnaire would then list a minimum of two and a maximum of five network members with whom respondents had reported the most contact. The questionnaire added variables measuring the relationship quality—most notably, the relationship difficulty—of a respondent with network members. The questionnaire further captured perceptual data on relationships between the network members, including their contact frequency and relationship difficulty. After completing this additional study, the data were linked to the main interview data to construct comprehensive personal networks. This additional study was carried out once, without follow-up.

The vast majority of eligible participants in the main interview (*N *= 1,193; 86%) agreed to receive an invitation to the additional network questionnaire, out of which *n *= 938 participants (79%) returned a completed questionnaire. We dropped respondents with missing values on any variables in the analysis. We also excluded 25 respondents who likely suffered from cognitive impairments based on a Mini-Mental State Examination score of 23 points or lower (see measures). The final analytical sample consisted of *n *= 892 respondents and their *n *= 4,273 network members. Respondents were, on average, 72.8 years old (standard deviation, *SD* = 7.3), and 52% were female.

### The *T*_2_ COVID-19 Additional Study

**Figure 1.**
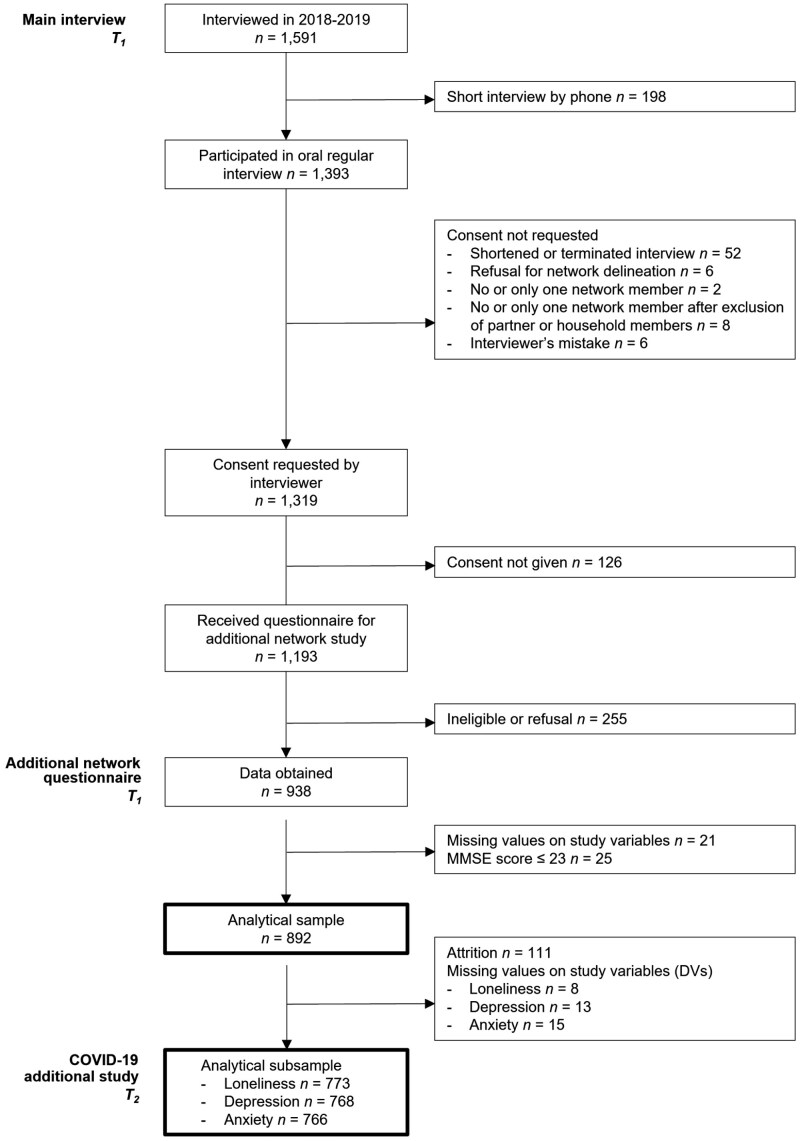
Flow diagram from interview sample to analytical sample. COVID-19 = coronavirus disease 2019.

As the COVID-19 pandemic presented a unique opportunity to measure mental health during a collective crisis, an additional study was added to LASA some months after the start of the pandemic (Hoogendijk et al., 2021). This COVID-19 Additional Study consisted of a postal or digital questionnaire on the impact of the situation around COVID-19 and several health variables. Mental health is typically reassessed only after every three years, so the shorter time lag for our outcome was a major advantage of this additional questionnaire. Data were received between June and October 2020. Out of 1,485 contacted LASA participants, 1,128 (76%) completed the additional study. After linking the *T*_1_ and *T*_2_ data, we arrived at analytical subsamples of *n *= 773 respondents for our longitudinal analysis on loneliness, *n *= 768 on depression, and *n *= 766 on anxiety. The selection of respondents across all studies is illustrated in Figure 1.

### Measures

#### Outcomes

Respondents’ mental health was assessed in the main interview at *T*_1_ and reassessed in the COVID-19 Additional Study at *T*_2_. *Loneliness* was measured by an 11-item scale (De Jong Gierveld & Kamphuis, 1985). An example of an item is “I miss having a really close friend.” Scale values are 0–11, with higher values indicating greater loneliness. *Depressive symptoms* were assessed with the short, 10-item CES-D scale (Radloff, 1977). With this scale, respondents indicate whether they had, for example, felt depressed or that everything was an effort in the last week. The scale’s values are 0–22; higher scores indicate higher depressive symptomatology. *Anxiety* was measured with the seven-item HADS-A subscale (Zigmond & Snaith, 1983). Respondents reported whether they had lately felt frightened or worried. This scale’s values are 0–17, with higher scores indicating more anxiety.

#### Independent variable

The presence versus absence of relationship difficulty in the respondent’s personal network was measured in the additional questionnaire at *T*_1_. The question reads, “Please indicate whether you think you have a good or a difficult relationship with these persons.” Its response options are on a five-point Likert scale ranging from “very difficult” to “difficult,” “both difficult and good,” “good,” and “very good.” *Relationship difficulty* was operationalized as present when one or more relationships with network members had been characterized by the first three categories (1), and otherwise as absent (0). This approach appeared feasible for three main reasons. First, within important and frequent contacts, purely negative relationships are naturally sparse, and the negative aspects inherent in ambivalent ties are likely more salient than positive aspects (Offer, 2021). Second, previous research has used name generators that ask respondents to identify individuals they “sometimes find demanding or difficult” (Offer, 2020, p. 422), thereby capturing partial difficulty in a manner similar to our approach. Third, the small number of (very) difficult ties did not permit a more fine-grained analysis of the first three categories.

#### Confounders

All respondent confounders were taken from the interview at *T*_1_. Sociodemographic variables were *age* (in years) and *gender* (1 = female, 0 = male), as well as variables assessed at *T*_*1*:_ the presence of a *partner* (1 = yes, 0 = no), the presence of *children* in the network (1 = yes, 0 = no), the respondent’s *educational level* (from 1 = elementary not completed to 9 = university education), and *employment status* (1 = employed, 0 = unemployed). *Self-rated health* measured whether respondents perceived their health overall as “excellent or good” (1) or “less than good” (0). A seven-item scale on limitations in *activities of daily living* (ADL) assessed the respondent’s performance in, for example, climbing stairs or taking a shower with or without difficulty and with or without help. Scale’s values are 1–5, with higher scores indicating more functional limitations. *Cognitive functioning* was measured with the Mini-Mental State Examination (Folstein et al., 1975), which has values ranging from 24 to 30; higher values indicate better cognitive functioning. *Self-esteem*, a measure of how individuals evaluate or appraise their own worth, was assessed by an adapted version of the Rosenberg self-esteem scale (Rosenberg, 1965). Scale’s values are 5–20, with higher values indicating greater self-esteem.

Several network characteristics were likely related to the independent variable, relationship difficulty in the network. All but the first network confounders were taken from the additional network questionnaire at *T*_1_. *Total network size* counted all generated contacts in the interview at *T*_1_. Notably, a minimum of two contacts was a prerequisite for participation in the additional network questionnaire. *Network density* represented the proportion of observed versus possible relationships between the members in the respondent’s network, excluding the respondent. We operationalized a relationship as being observed when two network members had been in touch and thus knew each other. Respondents were asked about every pair with the statement, “Please indicate how often you think these people are in touch with each other. Their contact may be in person, via telephone or internet (e.g., via email, WhatsApp).” *Network density* could range from 0 (empty, no mutual relationships) to 1 (fully connected, 10 relationships), with higher values indicating greater density. *Difficulty among network members* denoted the presence of any difficult relationship among the respondent’s network members, excluding the respondent. Respondents were asked about every pair with the statement, “Please indicate whether you think those persons have a good or a difficult relationship.” Answer categories were identical to the above question on relationship difficulty. The resulting variable was coded into the presence of one or more difficult relationships versus none.

### Analytical Procedure

We conducted our analysis on the respondent level and used treatment-effects estimation with *inverse probability weights* (IPW). This approach mimics an experimental study design that contrasts a potential outcome under two counterfactual scenarios (exposure vs. nonexposure to a treatment); it includes two estimation steps.

The first step resembles a logistic regression model wherein the probability of observing the treatment is regressed on the confounding variables. In this case, the treatment was regarded as our independent variable (i.e., the presence vs. absence of relationship difficulty in the respondent’s personal network) such that the confounders reflected the respondent’s and network’s characteristics. The resulting propensity scores were saved and used to calculate the IPW.

The second estimation step, conducted separately for the three outcome variables, linearly regressed these IPW on mental health. The resulting average treatment effect (ATE) denotes the mean difference in the mental health variables for respondents with and without relationship difficulty.

We employed IPW over a standard linear regression including all baseline confounders due to its higher precision with the treatment effect, particularly when a confounder correlates highly with the outcome (Raad et al., 2020). To further rule out endogeneity, we estimated models for prepandemic (*T*_1_) and peri-pandemic mental health (*T*_2_): Model 1 included the cross-sectional analysis of relationship difficulty and mental health at *T*_1_. Model 2a included the longitudinal analysis with relationship difficulty at *T*_1_ and lagged mental health at *T*_2_, adjusted for mental health at *T*_1_. This model excluded respondents without information on mental health at *T*_2_. That is, it used casewise deletion, causing a loss of statistical power. Model 2b was the same as the former model but included all respondents from Model 1. To this end, we imputed a missing mental health variable at *T*_2_ by taking a respondent’s previous mental health score at *T*_1_ plus the sample’s mean difference over time (*T*_2_ − *T*_1_).

Finally, we conducted a series of robustness checks to test the sensitivity of the results. All analyses were performed in STATA (18.0). A replication package with the code is available online (https://osf.io/j2kpr/?view_only=22fd949b0a3e42ce8e7bac31b12a741f); data are publicly available upon request (https://lasa-vu.nl/en/request-data/).

## Results

Table 1 presents the descriptive statistics (and, where applicable, the alpha) of all variables used in the analysis. Mean values for the mental health outcomes indicate higher loneliness, anxiety, and depression during (*T*_2_) than before (*T*_1_) the pandemic. Pearson’s correlation between the mental health outcomes (*T*_1_) was all statistically significant, with *r* = 0.328 between loneliness and anxiety, *r* = 0.442 between loneliness and depression, and *r* = 0.747 between anxiety and depression. A total of *n* = 135 (15%) respondents reported having one or more difficult relationships with members of their personal network. Out of those respondents, *n* = 60 reported difficulties with kin, *n* = 66 with nonkin, and *n* = 9 with both. More specifically, difficulty was most common with parents, siblings, and neighbors, as indicated by a mixed-effects logistic regression model adjusted for all confounders. These relationship types were followed by children-in-law and colleagues (although this was not a statistically significant association). Although few in number (*n* = 42 difficult relationships among *n* = 33 respondents), there are also older people who have close and difficult relationships with children.

**Table 1.** Descriptive Statistics, by Type of Variable

Variable	*T* ^a^	Alpha ^b^	Mean ^c^	*SD*	Min.	Max.
*Outcomes*						
Loneliness	*T*_1_	0.81	1.46	2.21	0	11
Loneliness	*T*_2_	0.83	3.03	2.86	0	11
Depression	*T*_1_	0.88	4.30	3.83	0	19
Depression	*T*_2_	0.82	5.71	4.04	0	22
Anxiety	*T*_1_	0.82	2.44	2.63	0	16
Anxiety	*T*_2_	0.82	3.23	2.93	0	17
*Independent variable*						
Relationship difficulty	*T*_1_		0.15			
*Confounders*						
* Respondent characteristic*						
Age	*T*_1_		72.8	7.3	61	100
Female	*T*_1_		0.52			
Has partner	*T*_1_		0.75			
Has child(ren)	*T*_1_		0.63			
Educational level	*T*_1_		5.0	2.1	1	9
Employment	*T*_1_		0.20			
Self-rated health (*ref.* = excellent/good)	*T*_1_		0.72			
Activities of daily living (ADL)	*T*_1_	0.84	4.7	0.5	1	5
Cognitive functioning (MMSE)	*T*_1_		28.4	1.5	24	30
Self-esteem	*T*_1_	0.72	15.8	2.1	5	20
* Network characteristic*						
Total network size	*T*_1_		17.5	8.6	2	36
Network density	*T*_1_		0.66	0.28	0	1
Difficulty among network members	*T*_1_		0.34			

*Notes*: CES-D = Center for Epidemiological Studies-Depression; COVID-19 = coronavirus disease 2019; HADS-A = Hospital Anxiety and Depression Scale - Anxiety; Max. = maximum; Min. = minimum; MMSE = Mini-Mental State Exam; *SD* = standard deviation. *N* = 892.

^a^ *T*_1_ = main interview and additional network questionnaire (years 2018–2019); *T*_2_ = COVID-19 Additional Study (year 2020). Loneliness (De Jong Gierveld) *T*_2_*n* = 773, depression (CES-D) *T*_2_*n* = 768, anxiety (HADS-A) *T*_2_*n* = 766.

^b^Scale reliability based on Cronbach’s alpha.

^c^For dummy variables, proportions only are reported.

The results from the first estimation step of the IPW model (the logistic regression model for relationship difficulty, our independent variable) revealed that relationship difficulty was more likely in unemployed respondents, those with lower self-esteem, a less dense network and who perceived relationship difficulty among their network members (results are presented in Supplementary Table 1). About one-third of respondents reported perceiving one or more difficult relationships among the members of their network. Notably, in an ordinary least squares regression model without adjustment—but not in a model with adjustment for confounders—relationship difficulty between network members was also significantly associated with higher loneliness and depression.

### Hypothesis Test

Table 2 displays the results from the treatment-effects estimation for mental health, the second estimation step of the IPW model. The potential outcome (PO) mean presents a mental health score for respondents without relationship difficulty. The ATE represents the difference in a mental health score for respondents with relationship difficulty compared to those without it. Hence, the average mental health score for respondents with relationship difficulty is retrieved by adding PO and ATE. Again, Model 1 estimated a cross-sectional association before the pandemic at *T*_1_, while Models 2a and 2b estimated a longitudinal association during the pandemic at *T*_2_. In the following, we discuss Models 1 and 2b, which used the identical full sample.

**Table 2.** Treatment-effects Estimation With IPWs for Three Mental Health Outcomes

Variable	Model 1^a^		Model 2a^b^		Model 2b^b^	
	Cross-sectional *T*_1_		Longitudinal *T*_2_		Longitudinal *T*_2_	
			(casewise deletion)		(imputed)	
	*N*	*B* (*SE*)	*N*	*B* (*SE*)	*N*	*B* (*SE*)
**Outcome: loneliness**	892		768		892	
ATE:						
Presence versus absence of relationship difficulty		0.493^*^		0.514		0.499
		(0.192)		(0.297)		(0.263)
PO mean:						
Absence of relationship difficulty		1.421^***^		2.972^***^		3.080^***^
		(0.079)		(0.108)		(0.101)
**Outcome: depression**	892		768		892	
ATE:						
Presence versus absence of relationship difficulty		0.995^*^		0.936		0.925^*^
		(0.392)		(0.527)		(0.420)
PO mean:						
Absence of relationship difficulty		4.168^***^		5.596^***^		5.865^***^
		(0.135)		(0.149)		(0.149)
**Outcome: anxiety**	892		766		892	
ATE:						
Presence versus absence of relationship difficulty		0.685^*^		0.375		0.316
		(0.277)		(0.445)		(0.342)
PO mean:						
Absence of relationship difficulty		2.343^***^		3.179^***^		3.267^***^
		(0.093)		(0.111)		(0.107)

*Notes*: ATE = average treatment effect; COVID-19 = coronavirus disease 2019; IPW = inverse probability weights; PO = potential outcome; *SE* = standard error. Mean for mental health outcome without relationship difficulty = PO mean, mean for mental health with relationship difficulty = PO mean + ATE.

^a^Relationship difficulty and outcome assessed in the main interview.

^b^Relationship difficulty assessed in the main interview; outcome assessed at follow-up in the COVID-19 Additional Study; model adjusted for previous outcome in the main interview.

^*^ *p* < .05. ^**^p < .01. ^***^p < .001, two-tailed test.

Our hypothesis predicted poorer mental health in older adults who reported any difficult relationships in their personal networks. Overall, the IPW models support this notion: All ATEs were positive, indicating that the presence of relationship difficulty was associated with higher scores in every model; associations were most pronounced in the cross-sectional models.

**Figure 2.**
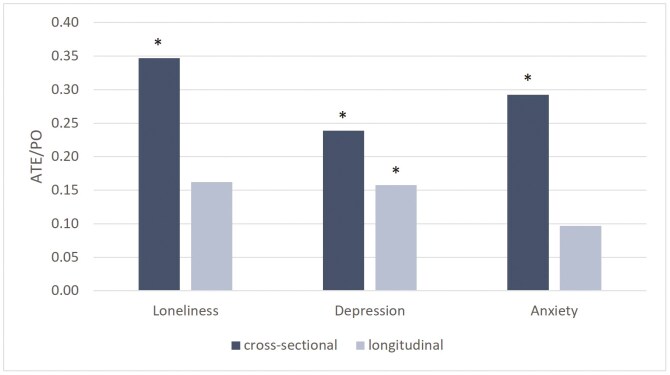
Average treatment effect (ATE) relative to potential outcome (PO) mean for three mental health variables. *N* = 892. Scales: loneliness: De Jong Gierveld; depression: CES-D; anxiety: HADS-A. Relative effect sizes are calculated as the ratio of the ATE and PO. Dark bars use the cross-sectional Model 1; light bars use the longitudinal Model 2b. CES-D = Center for Epidemiological Studies-Depression; HADS-A = Hospital Anxiety and Depression Scale - Anxiety. ^*^ATE significant at *p* < .05.

More specifically, respondents without relationship difficulty had an estimated loneliness score of about 1.4 points in the cross-sectional model and 3.1 in the longitudinal model. Their counterparts with relationship difficulty had an estimated loneliness score of about 1.9 and 3.6 points, which translates to an ATE of 0.5 points more in each case (1.0 and 0.9 for depression and 0.7 and 0.3 for anxiety, respectively). For all outcomes in the cross-sectional model at *T*_1_, and for depression in the longitudinal model at *T*_2_, the treatment effects were statistically significant. Depression thus showed the only significant lagged effect. The relative effect sizes (calculated as the ratio of the ATO and PO mean) are plotted in Figure 2. Sizes were largest for the cross-sectional model on loneliness at *T*_1_ and smallest for the longitudinal model on anxiety at *T*_2_.

Additional analyses showed that the ATEs deemed larger when considering difficult relationships with kin only (*n* = 60) as compared to difficult relationships with nonkin only (*n* = 66), suggesting a slightly greater impact of problematic ties in the family. We abstained from a more rigorous inspection and detailed interpretation by relationship type because the relatively rare occurrence of the treatment did not permit such fine-grained analysis.

### Robustness Checks

All robustness checks of the IPW models yielded results that are largely consistent with those produced by the original approach. We inspected robustness through several approaches. First, all but one covariate (gender: a standardized difference of − 0.13 after weighting) appeared well-balanced after weighting. Re-estimating the model without gender improved balance overall but did not establish substantially different treatment effects. Second, the IPW regression used the doubly robust method, meaning it was adjusted by the original set of covariates, to retrieve consistent estimators in case of model misspecification. The treatment effect for loneliness, however, dropped slightly and became insignificant. Third, the weights included extreme values near zero and one, so we retrieved bias-corrected confidence intervals through bootstrapping with 1,000 replications (Smith et al., 2021). Fourth, attrition in the COVID-19 Additional Study was higher among respondents with poorer mental health than those with good mental health. As imputation using the sample’s mean difference may have thus been biased toward a healthy score, we estimated a worst-case scenario (Jakobsen et al., 2017) using the mean difference plus 1 *SD*. In this way, imputed scores resembled below-average mental health. Fifth, as the outcome measures appeared quite skewed, we log-transformed them. Results overall yielded slightly larger effects, including significant ATEs for lagged loneliness. Sixth, since our analysis involved multiple testing across three correlated outcomes, we applied the Benjamini–Hochberg procedure with a false discovery rate of 0.05 to control for false positives. The resulting ranked *p* values confirmed the overall pattern of significance in our estimates.

## Discussion

The dark side of social networks has been acknowledged by researchers in health and aging (Cornwell & Schafer, 2016; Offer, 2021), yet empirical study designs often neglect that crucial aspect of certain social roles, such as children and caretakers. In our sample, 15% of older adults reported involvement in ambivalent or negative relationships. In line with previous evidence on psychological distress and loneliness (Chen & Feeley, 2014; Child & Lawton, 2020; Hua et al., 2021; Offer, 2020), our study lends support to the view that having difficult personal relationships is associated with higher loneliness, depression, and anxiety. Importantly, relationship difficulty showed a lasting effect on depression in our study: The presence of one or more difficult relationships in the personal network was related to greater depression more than one year later, even after controlling for prior depression scores. Since we controlled for total network size, our analysis indicates that the presence of positive relationships in the network did not completely offset harmful experiences from any negative encounters. Such findings existed independently of any other perceived difficult relationship in the network, even though relationship difficulty among network members was a strong predictor of personal difficulty with others. (Note that our data revealed no association between mental health outcomes and relationship difficulty among network members, possibly because conflicts among others are too indirect to have a measurable impact on respondents’ mental health.) As one to two out of 10 older adults experience relationship difficulty, and we operationalized relationship difficulty with a minimum of one troublesome contact, our study results have implications that are far from trivial.

Our finding that a significant number of older adults maintain difficult relationships appears to contradict a core principle of socioemotional selectivity theory (Carstensen, 1992, 2021). According to this theory, as people age, they prioritize emotionally rewarding relationships, and when such relationships are present, having a smaller network does not negatively affect well-being. However, our results suggest that certain close yet difficult relationships persist in later life, indicating that these ties may be normatively expected or necessary and, therefore, not easily eliminated—even if they negatively affect mental health. This is particularly relevant in relationships with adult children, where expectations of intergenerational solidarity and caregiving responsibilities can lead to an enduring mix of positive and conflict-ridden interactions, that is, intergenerational ambivalence (Gilligan et al., 2015), but also with other kin (Kyeremeh & Schafer, 2024).

Notably, the longitudinal effect sizes overall were smaller than the cross-sectional ones. Several explanations for this seem feasible. The longitudinal models controlled for earlier mental health, accounting for much of the statistical variance in later mental health; running the same models without such control or using a log-transformed variable (as outcomes appeared skewed toward healthier people) yielded larger effect sizes and even a significant association between relationship difficulty and loneliness. Moreover, during the time lag, negative relationships may have ended or become less important in the network such that they no longer led to lower mental health outcomes. In addition, the next available follow-up observation was organized during the pandemic, which we could not have foreseen when we organized the network data collection at *T*_1_, and which has implications for positive and negative relationships. Regarding the former, the pandemic was an unprecedented social crisis in which having good personal relationships is extra important for mental health. For such relationships, however, the stressful life event of the pandemic may have partially overshadowed the proposed mechanisms and altered the processes in personal networks: Daily routines and norms for social meetings changed abruptly and extensively, with critical implications for the handling and value of social relationships. Likewise, government-imposed social distancing rules justified and helped many avoid most face-to-face encounters, including hard-to-escape or difficult ones. Ambivalent and even negative relationships, by contrast, may have gained positive meaning during social abstinence, as simply having contacts available contributed to feelings of belongingness. In the peri-pandemic observation, however, it was impossible to collect comprehensive data on the network.

Related to this, we address the timing of the second mental health observation. The COVID-19 pandemic and related policy measures led to a global decline in mental health (Lau et al., 2023), including in the Netherlands (Van Tilburg et al., 2021), and contributed to smaller, more tightly knit social networks (Völker, 2023). While it remains unclear how the pandemic affected previously close yet difficult ties, these relationships may have either been excluded from downsized networks, reducing their impact on mental health, or become even more central, amplifying their negative effects. It is difficult to determine the pandemic’s influence without fine-grained time-series data, especially given the heterogeneity in both the extent and duration of mental health declines (Ellwardt & Präg, 2021). As a result, the pandemic may have simultaneously weakened and strengthened the association between relationship difficulty and poor mental health in our study.

The collection of additional network data, including in regard to the key independent variable (relationship difficulty), constitutes a significant contribution of our study to the literature. However, due to empirical sparseness and theoretical considerations, we combined difficult and ambivalent relationships in our analysis. While these relationship types share some characteristics, they may have distinct implications for mental health. We acknowledge the need for further research to disentangle their unique effects. Moreover, as we could only rely on a one-time measurement for relationship difficulty, our findings did not permit causal conclusions. Because our research design selected frequent contacts, difficult relationships with avoided others were underrepresented. This may limit the variability in relationship quality and potentially overlook the role of less frequent interactions. Having said this, in our data, contact frequency was only weakly correlated with relationship quality, indicating sufficient variation in relationship quality within the core network of the five most frequent, important contacts.

We thus recommend that future research follow difficult relationships with network members over time to assess the implications for mental health and possible feedback mechanisms on personal networks more precisely. Moreover, it would be interesting to apply a broader network perspective and explore the possible dynamics of mental health between members of the same network (i.e., to what extent connected older adults influence one another in their mental health). In this regard, previous research has suggested that individuals with similar mental health cluster in networks as loneliness and depression spread from one network member to another (Cacioppo et al., 2009; Rosenquist et al., 2011). Our data did not include information on the network members’ mental health. Similarly, ambivalence in family networks tends to involve third parties and a collective dimension (Girardin et al., 2018). Therefore, indirect connectedness to others’ ambivalent and imbalanced social relationships can also represent a source of stress in older adults (Ellwardt et al., 2020).

Ultimately, we believe that knowledge of the emergence and impact of potentially problematic network integration helps inform useful interventions for older adults’ mental health. In light of our stance and findings, we encourage social-epidemiological and sociogerontological researchers to undertake theoretical and empirical efforts to describe and explain commonplace relationships with others who are perceived as unpleasant or irritating.

## Supplementary Material

gbaf094_suppl_Supplementary_Figure_S1_Table_S1

## Data Availability

This study was not preregistered. A replication package with the code is available online (https://osf.io/j2kpr/?view_only=22fd949b0a3e42ce8e7bac31b12a741f); data are publicly available upon request (https://lasa-vu.nl/en/request-data/).
