# Supplementary material for: Relationship Difficulty Is Associated With Poorer Mental Health in Later Life
Source: J Gerontol B Psychol Sci Soc Sci. 2025 May 23;80(7):gbaf094. doi: 10.1093/geronb/gbaf094 (PMC12207867; doi:10.1093/geronb/gbaf094)
Supplement: gbaf094_suppl_Supplementary_Figure_S1_Table_S1 [file gbaf094_suppl_supplementary_figure_s1_table_s1.docx]

***The Journals of Gerontology, Series B: Psychological Sciences and Social Sciences*** **Supplementary Material: Ellwardt & van Tilburg. Relationship difficulty is associated with poorer mental health in later life.**


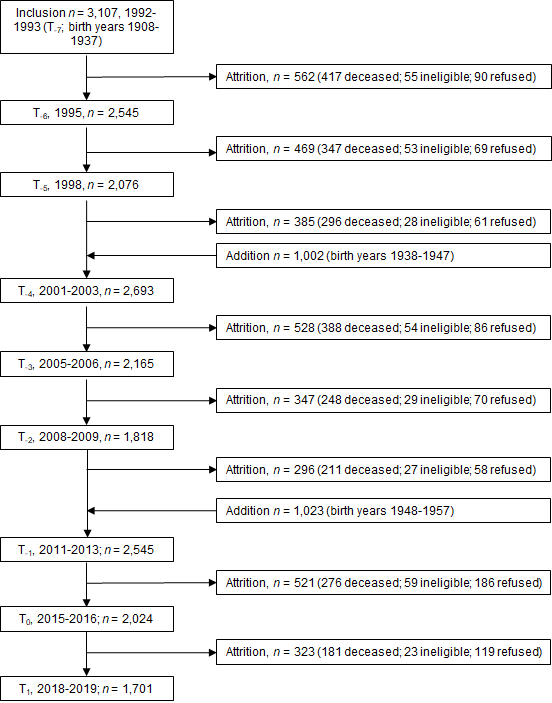


*Supplementary Figure 1*. Flow diagram on attrition in the interviews

*Supplementary Table 1*. First estimation step: logistic regression model on relationship difficulty

|  |  | Model S1 | |
| --- | --- | --- | --- |
| Confounder | Range | OR | CI |
| *Respondent characteristic* |  |  |  |
| Age | 61—100 | −0.0317 | −0.0658, 0.00253 |
| Female | 0—1 | −0.362 | −0.796, 0.0714 |
| Has partner | 0—1 | −0.329 | −0.813, 0.155 |
| Has child(ren) | 0—1 | 0.0841 | −0.363, 0.531 |
| Educational level | 1—9 | 0.0575 | −0.0484, 0.163 |
| Employment | 0—1 | −0.641^*^ | −1.232, −0.0503 |
| Self-rated health | 0—1 | −0.397 | −0.871, 0.0770 |
| Activities of daily living (ADL) | 0—5 | −0.201 | −0.601, 0.199 |
| Cognitive functioning (MMSE) | 24—30 | −0.0949 | −0.236, 0.0463 |
| Self-esteem | 5—20 | −0.137^**^ | −0.232, −0.0409 |
| *Network characteristic* |  |  |  |
| Total network size | 2—36 | −0.00117 | −0.0255, 0.0232 |
| Network density | 0—1 | −1.242^***^ | −1.965, −0.519 |
| Difficulty among network members | 0—1 | 1.681^***^ | 1.268, 2.094 |
| *Constant* |  | 6.769^*^ | 1.203, 12.33 |
| *N* |  | 892 | |

*Notes*. OR = odds ratio, CI = 95% confidence interval. ^*^ *p* < 0.05, ^**^ *p* < 0.01, ^***^ *p* < 0.001.
